# Association between beta‐blocker atenolol use and prostate cancer upgrading in active surveillance^1^

**DOI:** 10.1002/bco2.441

**Published:** 2024-10-09

**Authors:** Ali H. Zahalka, Ethan Fram, Evan Garden, Lauren Howard, Emily Wiggins, Mustufa Babar, Jay Annam, Allison Reagan, Benjamin Eilender, Amanda de Hoedt, Stephen J. Freedland, Ash Tewari, Kara L. Watts

**Affiliations:** ^1^ Department of Urology Icahn School of Medicine at Mount Sinai New York New York USA; ^2^ Department of Urology UT Southwestern Medical Center Dallas Texas USA; ^3^ Department of Urology Albert Einstein College of Medicine/Montefiore Medical Center Bronx New York USA; ^4^ Division of Urology Cedars‐Sinai Medical Center Los Angeles California USA; ^5^ Section of Urology Durham VA Medical Center Durham North Carolina USA

**Keywords:** active surveillance, atenolol, beta adrenergic blockers, beta adrenergic receptors, prostate cancer

## Abstract

**Objectives:**

The objective of this study is to investigate the association between the use of beta‐adrenergic antagonist atenolol and risk of pathologic upgrade in patients on active surveillance, considering growing literature implicating adrenergic innervation with disease progression mediated through beta‐adrenergic signalling.

**Patients and Methods:**

Men with low‐risk or favourable intermediate‐risk prostate cancer who were placed on an active surveillance protocol between 2006 and 2020 across three diverse urban hospitals were included. Exposure was duration of atenolol use, and outcome was pathologic grade group upgrading (to GG ≥ 3) on final prostate biopsy. Cox proportional hazard regression models were used to determine the associations between atenolol use and risk of upgrading with time, on a per‐examination basis.

**Results:**

A total of 467 men with initial GG ≤ 2 were included. Postdiagnosis atenolol use was associated with a decreased risk of pathologic upgrade to GG ≥ 3 on final repeat biopsy (HR 0.81, 95% CI 0.39–0.98). Longer duration of postdiagnosis atenolol use (>2 years) and greater cumulative atenolol dose (>730 defined daily doses) were associated with a more pronounced decreased risk of upgrade to GG ≥ 3 (HR 0.41, 95% CI 0.05–0.88, and HR 0.32, 95% CI 0.15–0.99, respectively). Initiation of atenolol use prior to prostate cancer diagnosis had a slightly greater protective effect than drug initiation postdiagnosis (HR 0.79, 95% CI 0.43–0.98, and HR 0.83, 95% CI 0.30–0.99, respectively).

**Conclusions:**

Beta‐adrenergic blockade with atenolol use in men on active surveillance is associated with a reduced risk for clinically significant grade group pathologic upgrade.

## 1 INTRODUCTION

Prostate cancer (PCa) is the second most common malignancy diagnosed in men, with most new diagnoses having localized disease.^2^ Men found to have low‐risk or favourable intermediate‐risk localized cancer on prostate biopsy are increasingly offered active surveillance as standard of care or preferred treatment.^3^ With time, more than a third of men experience pathologic upgrade, the majority of whom will pursue invasive treatment.^4^, ^5^ To prevent or delay invasive intervention in these men, multiple studies have assessed the association between pharmacologic agents and PCa.

Targeting adrenergic signalling in the prostate tumour microenvironment with beta‐adrenergic blockers, such as atenolol, for chemoprevention is a growing area of interest.^6^ Increased adrenergic nerve density is an early histologic hallmark of PCa development and a prognostic factor for disease aggressiveness.^7^, ^8^, ^9^, ^10^ Abrogation of adrenergic signals by genetic deletion of beta‐adrenergic receptors in murine PCa models inhibited cancer progression, as did pharmacological blockade with beta‐adrenergic antagonists/blockers.^9^, ^11^ These preclinical findings are supported by multiple large retrospective studies demonstrating a protective effect of oral beta‐blocker use on PCa risk.^6^, ^12^, ^13^

We have previously shown that among currently prescribed beta‐blockers, only long‐term atenolol use was associated with a significant reduction in incident risk for clinically significant intermediate and high‐risk disease on initial prostate biopsy.^6^ However, the association between atenolol use and sustainable protective effects on PCa progression has not been studied. Therefore, we examined the association between atenolol use and the risk of PCa upgrading on final repeat biopsy in men on active surveillance in a multicentre retrospective study.

## 2 PATIENTS AND METHODS

### 2.1 Study design and participants

**FIGURE 1:**
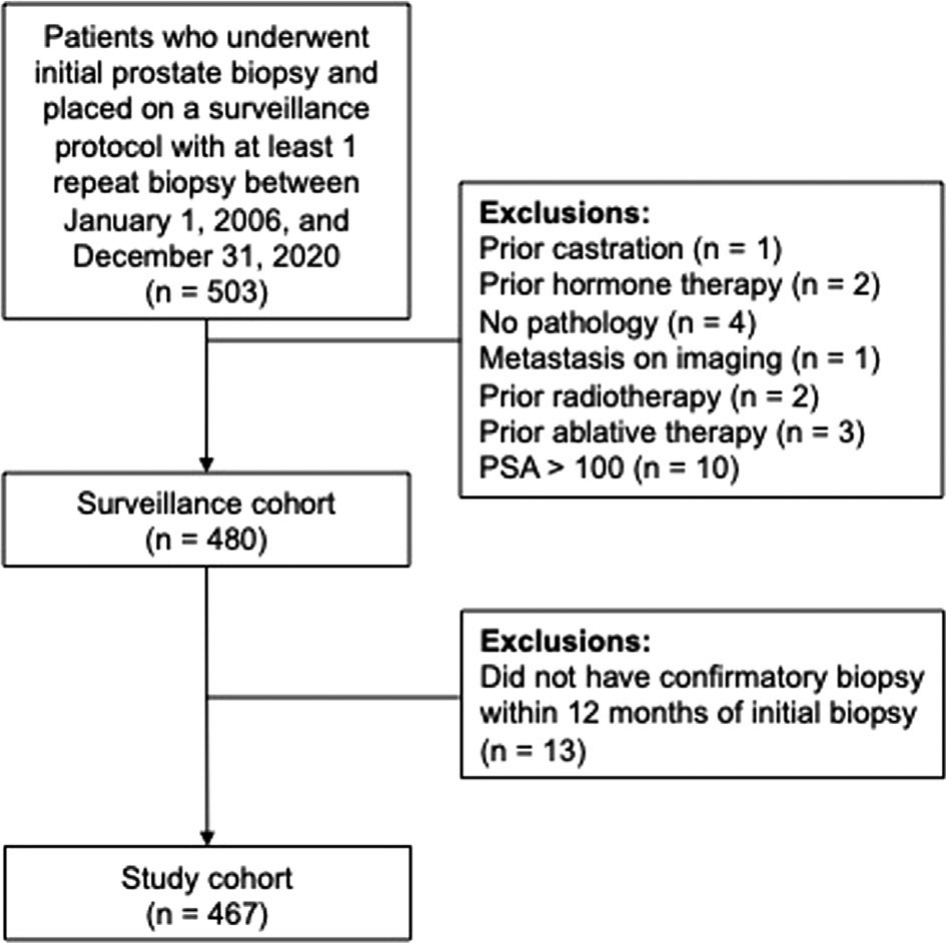
Patient selection flow diagram

This was an institutional review board‐approved retrospective study. Subjects were all men with clinical stage T1c or T2a PCa and an initial diagnosis of Gleason score 3 + 3 or 3 + 4 (GG1 and GG2, respectively) and were low or favourable intermediate risk by NCCN criteria^14^ who elected active surveillance at three large urban tertiary healthcare systems (Montefiore Medical Center, Mount Sinai Medical Center and the Durham Veterans Association Hospital) from 1 January 2006 through 31 December 2020, underwent a confirmatory biopsy within 1 year of initial biopsy and had one or more follow up biopsies thereafter. Serum PSA testing and digital rectal examination were done every 6 months. Pathological upgrading, which was the primary outcome, was defined as the appearance of Gleason primary Pattern 4 or greater or secondary Pattern 5 or greater on any follow up surveillance biopsy (GG ≥ 3). Patients who had either known or high clinical suspicion for metastatic disease (PSA > 100, MRI, CT, PET, bone scan or other imaging findings suggestive of disseminated disease), prior PCa therapy (radiation, cryotherapy and HIFU), underwent prior androgen deprivation therapy or whose data were incomplete for analysis were excluded from the study (Figure 1).

Patients' medical records were reviewed for biopsy characteristics including demographics, prostate specific antigen (PSA) level, prostate volume, digital rectal exam findings, family history of PCa, biopsy pathology and clinical staging at the time of biopsy. Atenolol use was determined by prescription refill history (Table 1) within the 5‐year period preceding the initial and repeat prostate biopsies. Atenolol users were defined as those with at least two prescription refills within the year preceding initial biopsy, and duration of use was calculated from medication initiation to last refill. Postdiagnosis atenolol use was expressed in the following three ways: ever use, cumulative duration of use and cumulative dose. Ever use of atenolol after PCa diagnosis was compared with never use up until the time of the final prostate biopsy. To determine whether there was a dose–response relationship between atenolol use and clinically significant PCa upgrading, two approaches were taken. Cumulative duration of use was defined as the total number of years of atenolol use calculated by summing the duration of all prescriptions between the initial and final prostate biopsies and then classified as less than 2 years or ≥2 years of use. To assess cumulative dose of atenolol exposure, atenolol use was expressed in units of defined daily dose (DDD), which is a validated measure determined by the World Health Organization for each medication.^15^ The DDD for atenolol is 75 mg, and cumulative dose was calculated by summing all DDDs from initial to final biopsy and classified as less than 730 or ≥730 DDDs.

**TABLE 1:** Characteristics of study patients

	Initial biopsy	Final biopsy
No. of pts	467	467
Median age, years	62 (58–68)	65 (60–70)
Median BMI, kg/m^2^	28 (25–31)	28 (24–31)
Median PSA, ng/ml	5.1 (3.0–7.7)	5.5 (3.0–9.1)
Median PSAD, ng/ml^2^	0.14 (0.09–0.20)	0.17 (0.11–0.30)
Median time on surveillance^a^, years	‐	3.0 (2.1–4.2)
Grade group (%)		
0	‐	74 (16%)
1	445 (95%)	160 (34%)
2	22 (5%)	99 (21%)
3–5	‐	134 (29%)
AUA risk group (%)		
Low	383 (82%)	269 (57%)
Intermediate	84 (18%)	148 (32%)
High	0	50 (11%)
CAPRA Score, Median	1 (1–2)	3 (1–4)
No. Beta‐blocker use, Atenolol (%)	85 (18%)	62 (13%)
No. race (%)		
Black	138 (30%)	
Caucasian	169 (36%)	
Hispanic	123 (26%)	
Other	37 (8%)	

*Note*: Data are median (IQR) or *n* (%).

Abbreviations: CAPRA, Cancer Prostate Risk Assessment; PSAD, PSA density.

^a^ Median time between initial and most recent repeat biopsy.

Because the Gleason grading system was updated in 2014, re‐classifying any cribriform or mucinous histology as Gleason Pattern 4 (previously scored as Gleason Pattern 3), we re‐reanalysed all pre 2014 pathology in our sensitivity analysis and excluded patients with cribriform or mucinous histology (naming this group pre‐2014 path adjusted).

### 2.2 Statistical analysis

Univariable analysis was performed using the chi‐square test for categorical outcomes and Wilcoxon Rank‐Sum for continuous variables. Cox proportional hazard models were used to test associations between atenolol use and risk of pathological upgrade to clinically significant PCa (GG ≥ 3), adjusted for potential confounders measured prior to cohort entry (race, GG on initial biopsy, BMI, age, PSA density, and cancer prostate risk assessment score, CAPRA), with data presented as hazard ratios (HRs) with 95% confidence intervals (CIs). In a secondary analysis, we determined whether prediagnosis use of atenolol acted as an effect modifier on the association between postdianosis atenolol use and risk of pathological upgrade to GG ≥ 3. All statistical tests were two sided with statistical significance set at *p* < 0.05. The R software environment (version 4.1.3) was used for all statistical analysis.

## 3 RESULTS

### 3.1 Patient characteristics

A total of 467 men met inclusion criteria for this study, with baseline characteristics listed in Table 1. Of these men, 445 (95%) had GG1 PCa on initial biopsy, and 22 (5%) had GG2 disease. Median PSA at initial biopsy was 5.1 ng/ml (IQR 3.0–7.7), and median time between first and most recent biopsy was 3.0 (IQR 2.1–4.2) years. At initial biopsy, 85 men (18%) were taking atenolol, 62 (13%) had active prescriptions at the time of final repeat biopsy and 99% of atenolol users were prescribed the medication for a cardiovascular indication. Of our multiethnic study population, 138 men (30%) were Black, and 123 (26%) were Hispanic. On final repeat biopsy, 74 (16%) had no cancer, 160 (34%) had GG1 PCa, 99 (21%) had GG2 disease and 134 (29%) GG3–5 disease (Table 1). Atenolol users and non‐users exhibited similar characteristics including a median of 2 (IQR 1–3) additional surveillance biopsies and median of 5 (IQR 3–7) PSA blood draws during surveillance, compared to a median of 2 (IQR 1–3) additional biopsies and a median of 5 (IQR 3–8) PSA blood draws in non‐users (Table S1).

### 3.2 Atenolol use and risk of pathologic upgrading

As the risk of pathologic upgrade on repeat prostate biopsy increases with time while on surveillance,^16^ we assessed whether atenolol use was associated with risk of clinically significant upgrade using Cox regression analysis. Atenolol use after PCa diagnosis (initial biopsy) was associated with a 19% decreased risk of clinically significant pathologic upgrade on final biopsy (Tables 2 and S2). Cumulative atenolol use and dose exhibited dose‐dependent relationships, with longer duration of use and higher cumulative dose associated with greater protection (≥2 years of use, HR 0.41, 95% CI 0.05–0.88, and ≥730 DDD, HR 0.32, 95% CI 0.15–0.99, respectively). We also assessed the effect of atenolol use prior to PCa diagnosis (prior to initial biopsy) on the association between postdiagnosis atenolol use and risk of upgrading. Prediagnostic atenolol use modified the association between postdiagnostic atenolol use and risk of PCa upgrading (Table 3); however, the difference was small (HR 0.79, 95% CI 0.43–0.98, and HR 0.83, 95% CI 0.30–0.99, for prediagnostic and no prediagnostic atenolol use, respectively).

**TABLE 2:** Multivariable cox proportional hazards analysis of postdiagnostic atenolol use and pathological upgrading on final biopsy

	Upgrade to clinically significant cancer^a^			
	Hazard ratio (95% CI)	*p* value	Adjusted hazard ratio^b^ (95% CI)	*p* value
No postdiagnotic atenolol use	Reference		Reference	
Postdiagnostic atenolol use	0.80 (0.51–0.98)	0.03	0.81 (0.39–0.98)	0.04
Duration atenolol use, years		0.04		0.03
<2	0.68 (0.16–1.05)		0.74 (0.43–3.26)	
≥2	0.39 (0.05–0.81)		0.41 (0.05–0.88)	
Cumulative atenolol dose, DDD		0.04		0.04
<730	0.84 (0.61–2.60)		0.87 (0.67–2.86)	
≥730	0.35 (0.13–0.99)		0.32 (0.15–0.99)	

Abbreviation: DDD, defined daily dose.

^a^ Clinicically significant cancer = Grade group 3, 4, 5.

^b^ Adjusted for race, BMI, age, PSA density, grade group 2 at first bx, CAPRA.

**TABLE 3:** Effect modification of prediagnostic use of atenolol on association between postdiagnostic atenolol exposure and pathological upgrading on final biopsy

	Upgrade to clinically significant cancer^a^		
	Atenolol use before diagnosis	No atenolol use before diagnosis	
	Adjusted HR^b^ (95% CI)	Adjusted HR^b^ (95% CI)	*p* for interaction
Postdiagnostic Atenolol use	0.79 (0.43–0.98)	0.83 (0.30–0.99)	0.04

Abbreviations: DDD, defined daily dose; HR, hazard ratio.

^a^ Clinicically significant cancer = Grade group 3, 4, 5.

^b^ Adjusted for race, BMI, age, PSA density, grade group 2 at first bx, CAPRA.

In 2014, there was a change in Gleason criteria that re‐classified prostate tissue samples with any cribriform or mucinous histology as Gleason 4 (rather than Gleason 3), thus resulting in a grade shift (many cases that formerly were graded Gleason score 6 became graded as Gleason score 7). As this change occurred in the middle of our study, we assessed the association between year of biopsy (pre‐2014 vs. 2014 and later) and risk of clinically significant GG upgrade and found no association (HR 1.04, 95% CI 0.71–1.53; Table S3). Due to the 2014 change in Gleason criteria, the post 2014 Gleason score 6 cases became a homogeneous group of tumours lacking cribriform or mucinous histology and had a less aggressive nature, in contrast to Gleason score 6 tumours of the pre‐2014 era. We therefore re‐reviewed the pathology of all initial biopsies performed pre‐2014 and excluded any samples with cribriform or mucinous histology. After excluding these samples, we found no association between biopsy year and risk of upgrade (HR 0.84, 95% CI 0.57–1.24; Table S3).

## 4 DISCUSSION

In this large, racially/ethnically diverse multicentre study, postdiagnostic atenolol use (a beta‐adrenergic receptor antagonist) was associated with 19% decrease in upgrading to clinically significant PCa on final repeat biopsy in patients on active surveillance. Furthermore, a protective duration/dose‐response in atenolol use was observed on pathologic upgrading with a 59% decrease in patients using atenolol for ≥2 years and 68% decrease in those consuming >730 DDD. This observation further supports previously reported findings that inhibition of adrenergic signals is associated with a decreased incidence, progression, recurrence and mortality from PCa.^6^, ^9^, ^12^, ^13^, ^17^, ^18^, ^19^

Multiple studies have assessed the association between pharmacologic agents and PCa with the hope of preventing or delaying invasive intervention in men on surveillance. Drugs targeting the androgen pathway have been some of the most promising. Dutasteride is a 5‐alpha‐reductase inhibitor used in the treatment of benign prostatic hypertrophy. In the reduction by dutasteride of clinical progression events in expectant management (REDEEM) trial, men with low‐risk PCa were randomized to dutasteride or placebo and followed for 3 years. While dutasteride use was associated with an increased chance of negative repeat biopsy compared to placebo, there was no difference in Gleason grade upgrading to clinically significant Gleason 7 or greater disease.^20^ These findings are generally consistent with the much larger Reduction by Dutasteride of Prostate Cancer Events (REDUCE) trial that found a reduced incidence of low‐grade PCa among men with a negative biopsy and follow‐up for 4 years with up to two repeat biopsies.^21^ However, unlike REDEEM, REDUCE found a slightly, but significantly, increased risk of high‐grade disease. Enzalutamide, an oral high‐affinity androgen receptor inhibitor used in the treatment of advanced PCa,^22^ was recently studied in the context of active surveillance. In the randomized study of Enzalutamide in Patients with Localized Prostate Cancer Undergoing Active Surveillance (ENACT), men with low‐risk or intermediate‐risk PCa who were in the enzalutamide arm were found to have a reduced incidence of pathologic upgrading at 1 year, but this difference was not sustained at 2 years follow‐up.^2^ Enzalutamide use not only has substantial financial cost but also exhibits a significant side‐effect profile including fatigue, increased adverse cardiovascular events and stroke.^22^ In contrast to anti‐androgens, atenolol is a beta‐adrenergic antagonist that is well tolerated and generic (off patent) and has more than 40 years of safety data demonstrating minimal long term adverse effects.^23^

Unique among beta‐blockers, atenolol exhibits pharmacodynamic properties that enable it to better penetrate the prostate.^6^ Atenolol is a polar molecule that attains a high plasma concentration, is renally cleared unchanged and bio‐accumulates in glandular tissue (such as the prostate) to supraphysiologic concentrations whereby it non‐selectively inhibits beta‐adrenergic receptors.^24^, ^25^ As both ADRB1 and ADRB2 (which code for the Beta1 and Beta2 adrenergic receptors, respectively) are highly expressed in PCa specimen,^1^ atenolol may have several mechanisms of cancer inhibition including acting on stromal beta‐adrenergic receptors to inhibit angiogenesis and tumour metabolism,^9^ as well as directly on PCa cells to inhibit migration.^26^ A recent triple‐blind placebo‐controlled Phase II study demonstrated that preoperative beta‐blocker use reduced expression of metastatic biomarkers and cell polarization in resected specimen from early‐stage breast cancer patients.^27^ While a small retrospective study suggested a mortality benefit of beta‐blocker use in high‐grade PCa patients,^19^ a recent Veterans Administration study did not find an overall survival benefit for beta‐blocker use in advanced PCa patients who were on concomitant androgen deprivation therapy.^28^ Taken together, these data suggest that the sweet spot for beta‐blocker use is early in the disease course, such as in patients with low grade cancers newly enrolled in active surveillance programmes.

A strength of this study is the size and ethnic diversity of our multicentre cohort, which enabled us to explore the association between atenolol use and pathologic progression in men who were followed on active surveillance. This study has several limitations. This is a retrospective study, which may introduce incomplete or inaccurate capture of data from each participating site. As mentioned above, there are pharmacologic agents, such as finasteride and statins,^29^ that may be potential confounders, and we did not control for due to limited data. Additionally, we were unable to account for fusion biopsies on repeat biopsy, as this information was not available for all sites. However, we excluded confirmatory biopsies done within a year of the initial biopsy to decrease the chance of upgrade due to under sampling, as previously described.^16^, ^30^ Furthermore, the clinical utility of fusion biopsies in active surveillance has recently been challenged on several merits as (1) the cumulative incidence of upgrading overtime has remained relatively unchanged in the MRI era^16^, ^31^; (2) MRI has not improved upgrading prediction models compared to those based on clinical factors alone^32^; (3) the majority of biopsy cores that result in upgrading to clinically significant cancer in surveillance patients come from the systematic biopsy cores rather than the MRI targeted cores^33^, ^34^, ^35^; and (4) in surveillance patients with stable MRIs, 20% exhibit progression from GG1 to ≥3.^36^ Another confounding includes a change in Gleason criterion that occurred in the middle of our study period (2014), potentially affecting risk of upgrading between the two eras. However, after accounting for this change by re‐review of the pathology, we found no association between biopsy year and risk of upgrade (Table S3). While a relatively small percent of patients were using atenolol, the large multicentre design of our study and the inclusion of diverse patient population provides us with insights into the protective association of atenolol and PCa progression. Ultimately, prospective randomized trials are needed to confirm these findings.

Long‐term atenolol use in men on active surveillance is associated with a reduced risk for clinically significant grade group pathologic upgrade over time. This suggests that atenolol may serve as a potentially beneficial adjunct to surveillance. Randomized placebo‐controlled prospective trials are needed to further assess this relationship.

## AUTHOR CONTRIBUTIONS

*Concept*: Ali H. Zahalka, Stephen J. Freedland, Ash Tewari, AND Kara L. Watts. *Data acquisition*: Ali H. Zahalka, Ethan Fram, Evan Garden, Lauren Howard, Emily Wiggins, Jay Annam, Allison Reagan, Benjamin Eilender, AND Amanda de Hoedt. *Statistical analysis*: Ali H. Zahalka. *Manuscript drafting*: Ali H. Zahalka, Mustufa Babar, Stephen J. Freedland, Ash Tewari, and Kara L. Watts. All authors have contributed to the final version of the manuscript.

## CONFLICT OF INTEREST STATEMENT

The authors declare no potential conflict of interest.

## Supporting information


**Table S1.** Characteristics of study patients by atenolol use.
**Table S2.** Hazard ratios of covariates included in Table 2.
**Table S3.** Cox proportional hazards analysis of the association between biopsy date and pathological upgrading on final biopsy.
