# Supplementary material for: Association between beta‐blocker atenolol use and prostate cancer upgrading in active surveillance
Source: BJUI Compass. 2024 Oct 9;5(11):1095–100. doi: 10.1002/bco2.441 (PMC11557265; doi:10.1002/bco2.441)
Supplement: Supplementary file 1 [file BCO2-5-1095-s001.docx]

Supplementary Table 1. *Characteristics of study patients by atenolol use*

	Atenolol use	No use
No. pts	85	382
Median age, years	64 (60–67)	62 (58–68)
Median BMI, kg/m2	28 (26–32)	27 (25–31)
Median PSA first biopsy, ng/mL	4.6 (3.3–6.5)	5.2 (3.0–7.7)
Median number of repeat biopsies, occurances*	2 (1–3)	2 (1–3)
Median PSA draws, occurances**	5 (3–7)	5 (3–8)
Grade group initial bx (%)		
1	77 (90%)	368 (96%)
2	8 (10%)	14 (4%)
3 – 5	0	0
No. race (%)		
Black	30 (35%)	108 (29%)
Caucasian	26 (31%)	143 (37%)
Hispanic	22 (26%)	101 (26%)
Other	7 (8%)	30 (8%)
Atenolol use, Indication		
Cardiovascular	84 (99%)	-
Migraine prophylaxis	1(1%)	-

Data are median (IQR) or n (%)

*number of repeat biopsies (bx) not inclusive of confirmatory bx within a year of initial bx

**number of PSA draws between initial bx and most recent bx

Supplementary Table 2. Hazard ratios of covariates included in Table 2

	**Upgrade to Clinically Significant Cancer***	
	Adjusted hazard ratio (95% CI)	p value
Race, Black	1.51 (0.89–2.56)	0.13
BMI, kg/m^2	1.06 (1.01–1.11)	0.01
Age, years	1.05 (1.02–1.09)	0.004
PSAD, ng/mL2	1.31 (0.55–3.11)	0.44
GG2 on intial bx	0.55 (0.16–1.86)	0.32
CAPRA score		
0–2	Reference	
3–5	1.86 (0.92–3.74)	0.08

*Clinicically significant cancer = Grade group 3,4,5

Supplementary Table 3. Cox proportional hazards analysis of the association between biopsy date and pathological upgrading on final biopsy

		**Upgrade to Clinically Significant Cancer***	
		HR (95% CI)	p value
**Initial biopsy date**	(Ref Post-2014)		
	Pre-2014	1.04 (0.71–1.53)	0.82
**Initial biopsy date** (Pre-2014 path adjusted)**	(Ref Post-2014)		
	Pre-2014	0.84 (0.57–1.24)	0.40

Abbreviation: HR, Hazard ratio

*Clinicically significant cancer = Grade group 3,4,5

**Excludes pre-2014 patients with cribriform or mucinous histologies on initial biopsy
